# “His Main Problem Was Not Being in a Relationship With God”: Perceptions of Depression, Help-Seeking, and Treatment in Evangelical Christianity

**DOI:** 10.3389/fpsyg.2022.831534

**Published:** 2022-04-19

**Authors:** Christopher E. M. Lloyd, Brittney S. Mengistu, Graham Reid

**Affiliations:** ^1^Human Sciences Research Centre, University of Derby, Derby, United Kingdom; ^2^Department of Global Health and Social Medicine, King’s College London, London, United Kingdom; ^3^Department of Experimental Psychology, Medical Sciences Division, University of Oxford, Oxford, United Kingdom

**Keywords:** Christian, evangelical, mental illness, depression, stigma, qualitative, story completion task, social perception

## Abstract

Some Christian communities may understand mental illness as the result of spiritual causes, such as sin, demons, or a lack of faith. Such perceptions are likely to influence how Christian individuals conceptualise and experience their mental health and enact help-seeking behaviours. This study explores perceptions of depression and mental health help-seeking in evangelical Christianity by using a novel qualitative story completion task. A convenience sample of 110 Christian participants from the United Kingdom completed a third-person, fictional story stem featuring a male with depression who entered his local church. A contextualist-informed thematic analysis illustrated how the disclosure of depression was represented as eliciting negative social reactions, potentially rendering individuals with depression as socially dislocated. Stories suggested that, increasingly, evangelical Christians may perceive a spiritualisation of mental illness, which negates reference to psychological, social, and biomedical representations, as unhelpful. Findings reveal the risks of a solely spiritual aetiology of depression and highlight how existing mental ill-health can be exacerbated if fundamentalist beliefs and approaches to therapeutic care are prioritised over holistic models of care. Methodologically, this study demonstrates the value of a rarely-used tool in psychology—the story completion task—for examining socio-cultural discourses and dominant meanings surrounding stigmatised topics or populations.

## Introduction

A growing body of research has shown that religious engagement may provide individuals with a sense of belonging, shared beliefs, and social identification, which may be associated with better mental wellbeing (Corrigan et al., 2003; Hayward and Krause, 2014). Religiosity can be defined as a multidimensional construct that refers to the variability in people’s commitment to some transcendental entity and related practices (Cohen et al., 2017). One of the ways in which religious engagement may serve this wellbeing benefit is through the provision of social support from like-minded believers who affirm the importance of the in-group membership (Sullivan, 2009; Starnino, 2016). Indeed, there is some evidence to suggest that involvement in religious life may provide an important social setting for adherents to develop coping strategies and worldviews, which lend themselves to better mental wellbeing (Hayward and Krause, 2014; Kinghorn, 2016). Taken together, religious engagement seems to be broadly associated with enhanced wellbeing outcomes, which may be partly explained by the inherently social nature of belonging to a religious group (Lloyd et al., 2021). In addition to the socially-derived wellbeing benefits, research has also shown that religious cognition is related to health outcomes (Page et al., 2020). Religion provides a framework for making sense of one’s existence and possible answers to life’s ultimate questions (Park, 2005). Studies have shown, for example, that religious struggles surrounding ultimate meaning are associated with the extent to which believers with depression experience therapeutic improvement (Currier et al., 2019). Looking at a spiritually integrated programme for individuals suffering from depression, Abernethy et al. (2018) found reductions in depressive symptoms from patients’ initial intake in the programme to their discharge. The authors also found that depressive symptoms were positively correlated with religious strain, whereas religious comfort was negatively correlated with participants’ level of depression. Interestingly, however, reductions in religious struggle over the course of the programme were not related to reductions in patients’ depressive symptoms. In contrast, the authors found that religious comfort was associated with a reduction in participants’ depression at discharge, suggesting a protective role of the positive aspects of religion in healthcare settings. In other research, religious comfort and struggle mediated the relationship between participants’ imagery of God at intake into a psychiatric programme and the change in their depressive symptoms at discharge. Currier et al. (2017) found that participants with positive relational imagery of God during initial inpatient assessments reported higher levels of positive affect at discharge in which the relationship was mediated by religious comfort. Looking at the results of these studies together it seems that the cognitive aspects of religion may confer some protection on believers’ mental wellbeing.

That being said, religion is not a monolithic construct, which can be persistently associated with positive effects on one’s mental wellbeing (Shadid et al., 2021). For example, research has suggested that in some Christian circles mental ill-health may be understood as the outworking of one’s inner spiritual condition (Webb, 2017). Indeed, there is some indication that for Evangelical Christians, discourse surrounding mental health may situate distress as emblematic of sin, demonic activity, or personal sinful behaviour (Dein, 2020; Lloyd and Waller, 2020; Lloyd, 2021a; Lloyd and Panagopoulos, 2022). With over 600 million global followers in which over 2 million are in the United Kingdom (Pew Research Centre, 2015; Evangelical Alliance, 2020), evangelicalism can be thought of as a Protestant transdenominational movement, which places an emphasis on the literal interpretation of biblical texts; the need for a personal saving relationship with Jesus Christ; and the expectation of spiritual health and wellbeing for believers (Bebbington, 1989; Stackhouse, 2007). Considering their global prominence and potentially spiritualised conceptualisation of mental illness (Lloyd and Kotera, 2021; Lloyd et al., 2021), there is clear need to better understand the Evangelical worldview and how this relates to perceptions of mental health and help-seeking behaviours.

Indeed, such an understanding of the perceptions of mental ill-health in Evangelical Christianity may reveal the importance of social influence on mental health help seeking and the emphasis placed on spiritual intervention in Evangelical communities (Wesselmann et al., 2015; Lloyd et al., 2021). By way of illustration, research has shown that the extent to which religious leaders endorse secular intervention seems to be associated with the help-seeking behaviours of their congregations (Cook and Hamley, 2020). This is important since whilst seeking religious social support may be helpful for some individuals, encouraging others with mental health difficulties to pursue exclusively spiritual interventions may be ineffective and at worse detrimental (Mercer, 2013; Asamoah, 2016). For example, encouraging evangelical Christians with mental health concerns to engage in frequent prayer as a form of treatment has the potential to prolong their distress if evidence-based interventions are not pursued in a timely manner (Keefe and Curtin, 2013; Lloyd, 2021a). Altogether, although religious participation has been associated with improved health outcomes in some studies, it is clear that the relationship between religion and health is not consistent. That is, that some religious communities may hold worldviews that preferentially favour spiritual over evidence-based secular interventions, which may have undesirable effects on people’s mental health (Lloyd and Hutchinson, 2022).

Of particular interest in the intersection between religion and mental health are experiences of depression. Depression can occur on a continuum, ranging from acute periods of distress to recurrent major depressive episodes, requiring psychosocial interventions (Patel, 2017). Though depression can be experienced in several stages of severity, it remains a common experience affecting almost 300 million people and representing the leading cause of mental health-related disorders across the globe (Herrman et al., 2019). Anti-depressants and psychological therapies present beneficial and efficacious interventions for depression; however, a large proportion of people with depression experience recurrent episodes which are to some extent treatment resistant (Hardeveld et al., 2013). Diverse experiences of depression reveal that the disorder does not always fit neatly within biomedical models of illness; instead, depression is both a subjective and socially located experience, where its trajectory is likely to be contingent upon broader social and cultural narratives of individuals and their communities (Haroz et al., 2017).

Bringing depression into the context of religious groups, it has been argued that understanding depression as the result of sin may pathologise depression as the individual’s choice (Scrutton, 2020). This conceptualisation of depression where the disorder results from wilful behaviours that are seen as unchristian means that treatments looking to increase one’s spirituality would be seen as efficacious. Indeed, research has shown that evangelical Christians may express heightened ambivalence toward secular treatments in which they question their suitability, preferentially endorsing faith-based interventions (Trice and Bjorck, 2006). For example, contemporary Christian self-help literature has described depression as a product of demonic influence (Webb et al., 2008), with other studies showing that over a third of Christians would prefer religious treatments for mental ill-health, including depression (Hartog and Gow, 2005).^1^

Yet despite the increasing growth of evangelical Christian denominations, in the United Kingdom and beyond (Leavey, 2010), we know little about how United Kingdom evangelical Christians perceive and respond to mental illness. Whilst the majority of previous research has quantified relationships between religiosity, spirituality, and depression (e.g., Ai et al., 2013; Balbuena et al., 2013; Braam and Koenig, 2019), or has taken place within a United States context, there is still a need to qualitatively capture perceptions of depression, particularly within a United Kingdom context. This is especially relevant due to the growing mental health debates within Christian communities that have been centred around theological and philosophical ontologies (see Scrutton, 2015, 2018; Swinton, 2015; Scrutton, 2020), which often omit societal perceptions and representations of mental ill-health. Researchers have either qualitatively captured lived experiences of depression and help-seeking (Martínez-Hernáez et al., 2014; Doblyte and Jiménez-Mejías, 2017) or mental wellbeing and its connection to Christian beliefs and practices. The philosophical groundwork laid by Scrutton (2020) warrants empirical research to contextualise how depression is represented within United Kingdom evangelical Christian communities and its influence on local discourses around mental ill-health and help-seeking.

In the current study, we aimed to use the novel story completion task (Clarke et al., 2019) to understand how evangelical Christians in the United Kingdom make sense of depression. Specifically, we were interested in understanding the intersections between depression and evangelical Christian faith through exploring how a (hypothetical) evangelical church community responds to a (hypothetical) male Christian who experiences low mood. The objectives of this research were to capture implicit individual perceptions and views of depression and the wider socio-cultural and religious discourses related to mental health help-seeking in evangelical communities. By focussing on evangelical Christians, this research sought to capture the connection between spiritual aetiologies and mental health help-seeking, revealing socially located assumptions, perspectives, and understandings of depression amongst evangelical Christians living in the United Kingdom.

## Materials and Methods

### Research Design

Data were collected using a story completion (SC) method, which is a novel qualitative method for accessing participants’ socially contextualised assumptions about a given topic (Clarke et al., 2019). SC has a long history in psychoanalytic (clinical) contexts (Rabin and Zlotogorski, 1981) where SC forms part of an intentional projective technique, where ambiguous stimuli are presented to access unconscious “truths.” Recently, SC emerged from the confines of psychoanalytic discourses and entered qualitative research (for a historical discussion of this qualitative method, see Moller et al., 2021). SC contrasts with direct and traditional self-report techniques, such as interviews or focus groups, which have dominated the field of qualitative research and have amassed numerous merits in their own right (Frith, 2013).

Clarke et al. (2019) state that the SC does not require participants to describe their experiences but rather imagine how a scenario would unfold in a particular context. This method encourages participants to complete a short story that responds to a first or third-person story cue, which is also the story’s opening sentence. As SC requires participants to draw upon the socio-cultural sense-making resources and repertoires at their disposal, this innovative method becomes a valuable technique for exploring implicit perceptions toward a given phenomenon. Because this method does not explicitly collect individual opinions or experiences, SC permits access to a range of meanings around the research topic, subverting the risks of collecting responses that are perceived to be socially desirable or immediately available (Clarke et al., 2017), which are both significant considerations when researching stigmatised contexts or marginalised groups.

### Theoretical Assumptions

Morrow (2005) recommends that qualitative researchers explicitly define the philosophical assumptions that influence their research. SC can be positioned in a varied range of epistemological contexts, extending from essentialist to social constructionist to contextualist. In an essentialist (i.e., realist) epistemology, analytic attention is directed toward defining psychological meanings thought to motivate stories, which demand personal motivations and feelings from the story writer. In a social constructionist lens, stories are not framed as capturing any singular psychological reality but rather are believed to reflect a discursive or relativist reality (Kitzinger and Powell, 1995; Burr, 2015). And in a contextualist epistemology, which lies somewhere between essentialist and social constructionist, stories are thought to reflect individual and social perceptions that are socially mediated and embedded (Moller et al., 2021; Lloyd and Panagopoulos, 2022). This study adopts a contextualist epistemology.

### Reflexivity

The notion of reflexivity, or reflecting upon one’s perspective, and how this might invariably shape and direct analytic interpretation, has a long history in qualitative research (Willig, 2012). Both CL and BSM were raised in evangelical Christian homes that emphasised charismatic spirituality and miraculous healing, whereas GR came to Christianity during early adulthood. CL is interested in the negotiation that ensues between faith, lived experience, and mental distress and how certain theodicies and theologies may influence individual and collective meaning making and wellbeing. He brings a range of intersectional standpoints and perspectives in relation to this research in that he is a Christian, psychologist, theologian, and scholar who is critical of theologies that demonise individuals or groups or prevent holistic help-seeking. BSM is a Global Mental Health researcher who investigates conceptualisations of mental distress and help-seeking behaviours in vulnerable communities. She has personally witnessed the complex relationship between evangelicalism and mental ill-health and has come to appreciate the need for researching and critiquing the nuances of mental health and religion. GR is a neuropsychological researcher interested in risk, resilience, and help-seeking for psychiatric conditions. He is a Christian with experience across different denominations who ascribe to differing views surrounding the issue of mental health and faith. All authors’ overlapping interests, experiences, and perspectives have allowed them to critically engage with the data through a motivating desire to investigate the potential impact of social and theological understandings of depression for evangelical Christians.

### Ethics

The procedures of the current study were approved by the Ethics Committee at the University of Derby (ETH2021-0074; see Lloyd, 2021b). All participants were fully briefed regarding the nature of the study prior to their engagement, with all participants providing informed consent. Given the online nature of the study, participants could withdraw their consent at any time by terminating their browser and up to 1 week following study completion. Data were anonymised and stored on a GDPR-compliant server to which the researchers had sole access. In light of the potentially distressing research topic, information pertaining to religiously sensitive mental health charities and helplines were provided in the debrief. All authors abided by the British Psychological Society’s Ethics Guidelines for Internet-mediated Research (The British Psychological Society, 2017).

### Data Collection and Analysis

Participants identifying as evangelical Christian, currently residing in the United Kingdom and aged 18 years or older were recruited to participate in this study. To assess their commitment to Evangelical Christianity, participants were asked to endorse creedal statements that aligned with Stanford and McAlister’s (2008) definition of Evangelicalism: (1) the Bible is the highest authority for what I believe; (2) it is very important for me personally to encourage non-Christians to trust Jesus Christ as their Saviour; (3) Jesus Christ’s death on the cross is the only sacrifice that could remove the penalty of my sin; and (4) only those who trust in Jesus Christ alone as their Saviour receive God’s free gift of eternal salvation.

Following ethical approval, individuals were recruited primarily through online Christian social media groups and subsequent snowball sampling between 2020 and 2021. A recruitment statement was posted onto group pages with the following text: “This study aims to explore perceptions of mental distress in the church community. Please consider taking part in this short, online creative study in which you will complete a short story. Anyone who is an evangelical Christian and lives in the United Kingdom is eligible to take part.” Participants were directed to Microsoft Forms to provide consent, basic demographic information, and to complete the story stem. All participants were provided with the following third-person story stem:

*“Tom had been extremely depressed and life has felt hopeless for a few years. Tom visited his local church where…’*

Participants were requested not to ponder but to provide their initial responses that captured “readily available” meanings and perceptions of depression. They were asked to dedicate a minimum of 10 min to completing the story, producing approximately 200 words or 10 lines. The SC was piloted with three individuals to ensure that participants understood the instructions and could engage with the study independently. A third-person story stem allows researchers to access less socially desirable responses, which might not otherwise be available through first-person accounts (Moller et al., 2021). These responses are captured by situating participants outside the story, where inferences of personal experience or viewpoints are often excluded (see Braun and Clarke, 2013). A single-story stem was provided to allow participants to go in-depth about the story, which could positively impact the length and quality of the story.

Participant stories were downloaded into Microsoft Word and imported into NVivo for data management and analysis. Data were analysed using Braun and Clarke’s (2006, 2013) approach to thematic analysis (TA), which includes six phases of coding and progressive theme development. The second author read and re-read all data, making initial notes, and analytic observations (phase 1). Systematic data coding followed, including isolating and identifying core features of the data (phase 2), which were then scrutinised for more prominent and recurring patterns of meaning to identify overarching themes (phase 3). Throughout this process, regular research analysis meetings between CL and BSM were used to further refine and solidify findings (phases 4 and 5). This manuscript constituted the final phase of analysis (phase 6), which incorporated selecting salient data extracts alongside theme definitions to produce a coherent analytic narrative. Spelling and typographical errors have been remedied in the excerpts of data provided, and participants have been assigned pseudonyms.

### Analysis Validity Procedures

Yardley’s (2008) quality measures for qualitative research were used to ensure research validity. Firstly, the researchers assessed their contextual sensitivity combined with an awareness of the existing research literature, ensuring that all data analyses were closely matched to the participants’ stories. This process was also supported with researcher theme validation meetings, whereby both CL and BSM discussed emerging themes and resultant disagreement. This helped to ensure that individual author’s biases were bracketed from undue influence upon the data analysis.

## Results

### Results Overview

A total of 110 participants provided a story, with the majority being female (*n* = 79) and aged between 21 and 75 years old (Table 1). The majority of the participants attended church several times a week (*n* = 32) or on a weekly basis (*n* = 59), and most participants had experienced mental health problems or had loved one with mental health conditions (*n* = 99). Participant stories ranged from 10 to 718 words (Mean = 183 words), consistent with published story completion work and indicative of data “quality” (Clarke et al., 2019).

**TABLE 1:** Descriptive summary of the participant demographics.

Characteristics		*n* (%)
**Age**		
	21–40	35 (31.8%)
	41–60	52 (47.3%)
	61–80	23 (20.9%)
**Gender**		
	Male	29 (26.4%)
	Female	79 (71.8%)
	Non-Binary	2 (1.8%)
**Church attendance**		
	Daily	3 (2.7%)
	Several times weekly	32 (29.1%)
	Weekly	59 (53.6%)
	Monthly	7 (6.4%)
	Seasonal	4 (6.4%)
	Yearly	2 (1.8)
	Never	3 (2.7%)
**Self-identify as evangelical**		
	Yes	91 (82.7%)
	No	19 (17.3%)
**Experience of mental illness**		
	Yes	99 (90.0%)
	No	7 (6.4%)
	Unsure	4 (3.6%)

Using a story mapping technique, which is a strategy that reveals common patterns and components of participants’ stories (Braun and Clarke, 2013), many responses followed a similar trend: (1) congregants welcomed Tom and offered him a sense of community and belonging; (2) Tom was invited to participate in Bible study groups, where he would eventually disclose his experiences of depression; (3) attempts of healing Tom’s depression led to spiritual growth or exit from the church. This linear trend in participant stories was more noticeable in succinct responses:

*“He was greeted with open arms, welcomed with cheering hospitality…and given a sense of belonging and family…He would hear a talk about someone who gives the ultimate hope and love no matter background, personal struggles and situations.”* (Anna, female)

Fewer stories (*n* = 11) described Tom’s receiving an unwelcoming or mixed response from congregants, where individuals with depression were made to feel invisible against the backdrop of social interactions before church services. One story mentioned, “*It was very busy around him with people chatting in groups, clearly all already knowing one another. Tom felt even more alone than he normally did*” (Gabby, female). Hesitations in making oneself visible were compounded by perceptions of congregants, who were often described as “*happy*,” “*fulfilled*,” “*smiley*,” and additional adjectives that painted a positive imagery of evangelicals. These stories illustrate how individuals with depression may perceive religious social interactions as challenging to navigate:

*“This was hard to sit through for Tom, surrounded by people who just talked about how great everything was made him feel bad about how sad he was”* (Lucy, female).

Many stories (*n* = 33) described the importance of spirituality and faith in addressing depressive symptoms, whereas a smaller sample of stories (*n* = 23) described depression as having a biological component. The latter stories emphasised the importance of seeking formal mental health care from a GP or through counselling. One participant described, “*he decided he had to try the doctor who recognised the symptoms right away, prescribed some medication, and recommended some counselling and CBT*” (Thomas, male). Other stories (*n* = 17) described how the decision to seek help was aided by social support networks within and, at times, outside of the church. Where clinical approaches to depression were mentioned, Tom’s story always concluded with a happy ending, describing a reduction in depressive symptoms, increased confidence, or becoming a mental health advocate.

However, not all stories ended adaptively, as the prevailing perceptions of treating depression were sometimes driven by spiritual interventions. The following sections pay closer analytic attention to how depression is represented within the evangelical church. After the disclosure of depression, it appeared that normative discourses constructed individuals with depression as problematic, which is reinforced through external and internalised stigma. Experiences of stigma then influenced social interactions within the evangelical church. We describe two commonly mentioned modes of healing (spirituality and sociality) and its connection to perceptions of curing and treating depression. We conclude this section by describing perceptions of failed expectations of healing and offer a critique on how the pressure to experience miraculous healing may exacerbate mental ill-health and push individuals out of the evangelical church. As a whole, this section describes the perceptions of depression and help-seeking amongst evangelical Christians by analysing stories that underscore social reactions to Tom’s lived experiences of depression and the varied modes of treatment.

### Problematising Individuals With Depression

In several stories (*n* = 18), the social reactions to Tom’s disclosure of depression revealed how mental ill-health was problematised within the evangelical church. The problematisation of depression appeared to be grounded in normative spiritual aetiologies: a phenomenon that is characteristic of evangelical Christian perceptions of mental distress (Lloyd, 2021a). The construction of depression as problematic emerged in conversations with congregants, which simultaneously reduced depression to a spiritual deficit:

“*He was told his main problem was not being in a relationship with God. (Logan, male; emphasis added) [We] helped him understand there was a way forward with God that would rid him of his problem.*” *(Lyla, female; emphasis added)*

Stories problematising Tom revealed how depression was understood as an elusive, temporary condition that could be quickly and easily cured by spiritual remedies, like building a relationship with God. In some cases, these distinctions also differentiated physical from mental ill-health. For example, one participant wrote:

“*They told him to snap out of it but never said anything similar to people with physical disabilities or health problems.*,” *(Maria, female).*

Deviating from the SC task, one participant who indicated attending church several times a week critiqued the presence of a mental health discourse in the evangelical church, revealing how the problematisation of depression was deeply intertwined with theological beliefs:

*They said, “Often, people, these days are depressed because they are not getting something they do want (materially or experientially) or they are getting something they don’t want” (Edward, male).*

He continued by arguing that in many cases, depression is “*illegitimate*,” adding:

*“Depression does not automatically make you a victim. In this instance, the hope of the gospel should be able to lift him out of his despair if he is able to believe it, take responsibility for the things he might have done wrong and repent.”*

By comparing depression to victimisation, this participant situated depression as an experience that occurred because of one’s wrong behaviour, thus requiring ownership, reconciliation and heightened spirituality to overcome mental ill-health. Such stories parallel Scrutton’s (2020) view that solely spiritualised aetiological accounts of mental distress overemphasise the role of individual responsibilities, whilst minimising its social and relational context. Furthermore, this construction of depression may reinforce notions of mental ill-health as antithetical to Christian faith, thus requiring a spiritually-oriented treatment. This interpretation of mental ill-health eschews secular conceptualisations for a theological interpretation (Wesselmann and Graziano, 2010). Stories suggested that the problematisation of depression also influenced how congregants interacted with individuals experiencing depression for an extended period. Though the majority of stories (*n* = 39) began with Tom’s being welcomed into the church, social relationships had the potential to negatively change if Tom’s depression did not dissipate:

*“When he tried to get more involved with the church*…*his mental illness was seen as a real problem in the selection process. Soon Tom was seen as a bit of a problem, and leadership no longer really wanted him involved in things. There was an attitude of ‘thus far, but no further, as really you’re broken” (Nathan, male; emphasis added)*

Social interactions that problematise mental ill-health can influence an individual’s self-perception, where the notion of being problematic becomes internalised as self-stigma (Corrigan, 2004). Self-stigma within the evangelical church is not uncommon as studies have shown evangelical Christians to display higher degrees of self-stigmatised depression than non-Christians (McGuire and Pace, 2018). The connection between the external and internal stigmatisation of depression emerged in participants’ stories as internal dialogic self-reflections, where Tom would contemplate the cause of his depression and his comfortability in opening up to fellow congregants. In some stories (*n* = 13), Tom worried about congregants’ reactions to his experiences:

*“He was scared that if he was honest about his difficulties people would avoid him and disregard him. He was embarrassed by his depression and kept it secret” (Amber, female).*

Experiences of self-stigma were also illustrated through descriptions of depression’s being “*embarrassing*,” where Tom lacked “*self-confidence*” and resorted to self-blame and shame as a means of withholding his experiences of depression.

The problematisation of individuals with depression revealed how stigma could be experienced and perpetuated within the evangelical church and how disclosure could render depression visible. The visibility of depression appeared to garner similar reactions as the (visibly) disabled community, where their differences have primarily been considered troublesome within Christian churches (e.g., Eiesland, 1994; Creamer, 2012; Reynolds, 2012). Though congregants often perceived the prognosis of physical and mental ill-health to be different, the stigma and treatment of individuals with depression as “*broken*” amplified the visibility of individuals with mental ill-health and subsequent approaches to treatment. As disability theologians and scholars have noted (Eiesland, 1994), the problematisation of visible impairments is accompanied by a perception that “problems” should be fixed or cured, thus drawing attention to how Christian communities perceive remedies to various illnesses. The following sections explore representations of common spiritual remedies and reveal how a theology of healing has dominated informal treatments of depression.

### Healing Through Social Connection

Many stories (*n* = 58) stressed the importance of social support in alleviating Tom’s depressive symptoms. By describing how Tom was invited to converse and develop relationships with congregants, stories revealed how evangelical Christians perceived interpersonal relationships as preferred modes of providing emotional, social, and spiritual support and a remedy for depression. By describing Tom as sitting alone in the back of the church, several stories (*n* = 15) situated the experience of depression as connected to social isolation, revealing how social connection was positioned as the principal route to recovery. Moments of social contact typically began when congregants noticed Tom sitting alone in the back of the church and invited him to converse:

“…*a middle-aged man sat near him and simply chatted, asking Tom a few non-invasive questions and introducing himself. Then bought Tom a cup of tea and a biscuit. He didn’t evangelise or get pushy, but he did try to make Tom feel welcome.” (Harrison, male)*

*“People noticed he was unhappy and didn’t seem to know anyone. So, a kind couple invited him to their house after church for lunch. They didn’t want to intrude, but they made it clear they would be there for him*…*” (Oscar, male)*

In some stories (*n* = 25), these moments of social interaction developed into relationships that encouraged Tom to join Bible study groups. Descriptions of Tom’s receiving invitations to develop relationships with congregants highlighted how Bible study groups were perceived to be a therapeutic resource. Though most research on social support in religious settings has routinely conceptualised its dimensions to include emotional, informational and instrumental (Merino, 2014; Salusky et al., 2021), exploring the spiritual dimension of sociality elucidates the nuances of support in religious settings. Spiritual social support like prayer and reading the Bible are perceived as ideal types of support an evangelical Christian can offer individuals experiencing depression (Wesselmann et al., 2015). Stories suggested that individuals with depression may feel accepted and supported in these settings after disclosing their experiences within a trusted social group. For example, one story succinctly said:

*“As he got to know people, he shared more of his life and they offered him practical help and moral support,” (Riley, female).*

Another story echoed similar sentiments, directly challenging the problematisation of individuals with depression:

*“It was a relief to Tom to feel accepted, and to be considered a person, rather than to be treated as a problem to be solved.” (Bella, female).*

In stories where social support was mentioned, the concept of healing often followed, frequently being described as increasing positive attributes (e.g., hope, confidence, and calmness) and decreasing depressive symptoms. For example, one story mentioned, *“He met a group of Christians who uplifted him and supported him through his trials and tribulations. Very soon, Tom slowly began to come out of darkness and was filled with hope, love and patience” (Cody, male).* However, one story explicitly connected sociality to healing: “…*where he was made welcome and was encouraged to be part of a life group and eventually healed from his depression as he was able to experience God’s love” (Nancy, female)*.

Though this last statement omits the complexities around help-seeking and social reactions to individuals with depression, it reveals the dangers of a reductive perspective on addressing mental health issues within evangelical settings. By explicitly connecting “experiencing God’s love” to miraculous healing, individual experiences of depression risk being minimised to an acute experience that is overcome by spiritual social support.

In some stories (*n* = 18), sociality became a bridge to spirituality, where individuals developed a greater sense of “*faith*” because of their interpersonal relationships and involvement in Bible study groups. For example, one story mentioned, “*He made some great friends who got alongside him, helped him to explore biblical hope. Over time he gave his life to Jesus and, alongside medical support, was able to claim he was free from depression*” *(Tommy, male).* While social interaction is important for alleviating experiences of depression, these excerpts reveal that the emotive element is key to enhancing individual mental wellbeing, as it provides the “comfort of knowing that one has support” (Hovey et al., 2014, p. 387). Additionally, the spiritual component of social support is also widely supported amongst evangelical Christians because of the dominant spiritual aetiology that is primarily attributed to the cause of mental ill-health (Wesselmann et al., 2015).

Though not all stories that centred sociality ended with a reduction in depressive symptoms, they illustrate how the long-term effects of social interactions were considered a conduit of healing grounded in ideals of fellowship, where spiritual and emotional social support could aid individuals experiencing depression. The following section further explores the perceptions of spirituality as a mode of healing.

### Healing Through Spirituality

Despite the story stem’s omitting Tom’s religious affiliation or spiritual background, most stories (*n* = 59) described him as areligious and in spiritual deficit. These stories alluded to how individuals with depression should become more spiritual as a more intimate relationship with God would provide healing from depression. In this context, Christian spirituality, or the pursuit of a relationship and connection with God (Koenig et al., 2012) becomes an avenue for healing. These perceptions are similar to the experiences of disabled Christians (Clifton, 2014), illustrating how attaining healing has become an automatic response to curing individuals of (in)visible ailments. Though prayer is often described as a primary mode of healing (Clifton, 2014), participant stories also listed conversion, church attendance and additional practices that constitute increased faith or belief in miraculous healing:

*“They offered to pray with him and suggested he attend a [conversion] course*…*they would encourage him to come to church on Sunday*… *May offer ‘prayer counselling’ a form of quasi counselling delivered by respected members of the community but who are not necessarily qualified counsellors.” (Jessica, female)*

*“The Minister tried to convert him and assured him that once he became a Christian his depression would leave him for good. And encouraged him to come to Church regularly. The Minister wanted to lay hands on Tom to drive out the sickness of his mind.” (Arthur, male)*

Though the emphasis placed on healing reveals elements of spiritual reductionism, participants’ stories also revealed how the relationship between healing and spirituality was perceived as much more complex and nuanced. These stories revealed that heightened spirituality and religiousness, or the adherence to religious practices (Koenig et al., 2012), can be achieved through multiple avenues, occurring independently. However, the perceived relationship between spirituality and healing was clear: heightened spirituality was the precursor for healing.

These perceptions about healing also reveal the interplay between social support and theological beliefs, where social interaction precedes the spiritual encounters necessary to experience miraculous healing. A few stories (*n* = 9) detailed Tom’s piqued curiosity in Christianity and interest to return to church the following Sunday, and some stories (*n* = 11) described his depressive symptoms slowly dissipating due to heightened spirituality:

“…*he learnt more of the Lord, His truths and promises along with understanding more of scriptures*…*this gave him a sense of hope, raising his spirits*…*Over time, Tom was able to share with others how [the bible study] group and the Lord, helped him to overcome his depression.” (Clara, female).*

Heightened spirituality did not always lead to healing, however, as very few stories (*n* = 6) described the possibility of becoming more spiritual and experiencing depressive symptoms simultaneously. For example, one story concluded, “*At the end*…*Tom gave his life to the Lord. His troubles were not over but he knew the Lord walks with him*…” *(Zoe, female)*. *While another one read*, “…*he still had mental health issues but now he felt God was behind him, calling him back from the dark abyss*” *(Dylan, male).*

Though stories revealed a dominant perception that healing occurs through spirituality facilitated social interactions, a smaller sample of stories revealed the harms associated with expectations of healing. The following section outlines how perceptions of prolonged experiences of depression, despite attempts of healing, may worsen mental ill health and create the conditions for exiting the evangelical church.

### The Failure to Heal

The inability to receive miraculous healing for depression was routinely phrased as Tom’s failure and lack of spirituality to obtain healing. This perception further problematises individuals with depression, having the potential to cause significant harm to an individual’s mental health. Like one story mentioned, “*He was encouraged to pray more and trust God more. Tom felt that he had failed as a Christian as well as failed in life. However much he prayed, he still felt depressed” (Ruby, female).* Generally, individuals with depression may struggle to complete or engage with everyday tasks (Fuchs, 2014; Stanghellini et al., 2017), but when performing religious acts are predicated on receiving miraculous healing, the inability to heal may compound existing experiences with mental ill-health. For example, one story described Tom’s receiving prayer and being told that Jesus would heal his depression. After not receiving immediate healing, Tom was encouraged to “press in” by reading the Bible and praying more frequently. The story continues:

*“Tom tried, he really did, but with his depression, getting up was hard enough anyway, forget leaving time to read his Bible too. He did pray, but that seemed to become more about feeling anxious because he hadn’t read his Bible that day and because he didn’t seem to be praying the right way to be healed. Other friends in the church suggested he just needed to “have more faith,” but no one ever seemed to say what that meant or how you would go about it. Now he was feeling guilty about that too. He began to avoid church meetings and people. He just couldn’t face having to tell them their prayers hadn’t been answered AGAIN.” (Eleanor, female)*

Feelings of anxiety, guilt, loneliness, and intensified depressive symptoms were mentioned as outcomes of failed attempts to heal, consistent with Dein’s (2020) assertion that the inability to strictly adhere to religious performances could escalate experiences of anxiety and depression. These experiences can potentially exacerbate existing mental ill-health despite individual efforts to “*press in*.” “Pressing in” emerged as a concept related to religiousness and spirituality; however, this idea was abstract for individuals unfamiliar with evangelicalism. For example, one participant mentioned, *“They told him if he “pressed into God” he would feel better, but he didn’t really know what this meant.” (Grace, female).* The disconnect between religious-informed care and individuals unfamiliar with a theology of healing reveal how its performance can be unclear and poorly defined. Here, it is important to consider Clifton’s (2014) critique of healing ministries. Clifton argues that the inability to experience supernatural healing negatively impacts people who are not healed, resulting in disappointment, confusion, anger, and guilt. These experiences and expectations of healing cause individuals with (in)visible disabilities to feel uncomfortable attending churches who embrace healing theologies. Though Clifton’s argument centres experiences of the disabled community, the relationship between healing and distress are also applicable to individuals experiencing depression. Participant stories revealed that people who enter the evangelical church in search of belonging and support may feel more isolated and unwelcomed when their experiences do not conform to the normative ideological expectations of recovery and healing.

The inability to achieve miraculous healing through sociality and spirituality often resulted in Tom leaving the church: *“Tom realised that he did not have the personal resources to be able to be a useful member of the congregation*…*this compounded his depression and feelings of hopelessness, and Tom made the decision to leave the church” (Alice, female).* Stories of departure revealed how the stigma of mental ill-health and failed healing created negative social interactions that isolated individuals with depression, where they eventually sought support outside of the evangelical church. In these stories (*n* = 10), Tom either joined a non-evangelical church, sought support from the medical community or left the Christian faith. However, three stories described a permanent, more intense exit from the evangelical church – suicide.

Though a suicidal outcome constituted a fraction of stories, it is essential to note that this form of exit, from the church and life, may be a reality for individuals experiencing depression. As one story described:

*Tom soon began to notice his community avoiding him as without miraculous healing they thought that he was not a “true” Christian. Still, Tom tried his best and soon stopped talking about his struggles. People were more accepting and welcoming of him that way but it took its toll and his depression got worse and worse. One Sunday night, after an evening service, he couldn’t take it anymore. Why had God made him this way and why was he not helping? Why did his community ostracise him when he talked openly about his struggles? Tom took an overdose and died. His community blamed it on the devil and him not having enough faith to get through. (Skyler, non-binary)*

Christian perceptions of reasons individuals take their own lives range from hopelessness, despair, social disconnect, depression, lack of support, and not believing in Jesus (Bazley and Pakenham, 2019). Though several of these factors emerged in participants’ stories, the role of healing and problematisation of mental ill-health has not been considered in research on suicide in Christian churches, especially in evangelical communities. Presently, research has shown that increased religiosity correlates with low suicidality in Christian communities (Bonelli and Koenig, 2013; Stack, 2018). Participants’ stories presented here illustrate how the pressure to experience miraculous healing for depression may exacerbate experiences of mental ill-health, including the possibility of suicide. This dominant perception on the role of healing for depression reveals it to be cyclical where failed expectations of receiving a cure subsequently render individuals problematic.

## Discussion

The aim of the current study was to explore religious discourse and the wider socio-cultural perceptions of depression and help-seeking in evangelical Christians in the United Kingdom. To this end we used a novel story completion task to understand how evangelical Christians constructed and made sense of depression by asking participants to write a short narration of how a hypothetical individual with low mood would be received by a hypothetical Christian community (Clarke et al., 2019). This third-person story stem allowed participants to deflect their personal experiences of mental ill-health in which participants crafted expressive stories about depression, help-seeking, and treatment that revealed the interconnectedness and complexities of seeking psychosocial support within evangelical communities. Depression was frequently represented in stories as being viewed as a spiritual deficit, where individuals were rendered responsible for their experiences of depression. Whilst this finding aligns with earlier research, such as Scrutton’s (2020) “sin account of depression,” and other qualitative data (Lloyd, 2021a; Lloyd and Hutchinson, 2022), there is also some nuance. Specifically, data from the present study suggests that increasingly, evangelical Christians may recognise the dangers of a solely spiritualisation of psychological distress, oftentimes recognising that this may create a climate of stigma. In this regard, many stories positioned solely spiritual solutions (prayer, deliverance, and healing) as leading to negative conditions for individuals with depression, including failed expectations, stigma, and marginalisation.

Analogous to this, healing through sociality and spirituality seemed to be the preferred and primary mode of treatment, often being prioritised over biomedical models of therapeutic care. While some individuals may experience improvements to their mental health, failed expectations of care remain a reality for others, exacerbating their mental-ill health, heightening their visibility, and solidifying their positioning as problematic within their community.

The fear of disclosing experiences of depression became apparent in stories where the internal dialogic self-reflections illustrated a paradox of help-seeking within the evangelical church. In these stories, the longing for social contact that would render depression visible contrasted feelings of invisibility and loneliness. As a result, the disclosure of depression inadvertently exposes an individual’s mental health needs and negatively alters social interactions that reinforce discourses that individuals with depression are problematic or spiritually inadequate. Disclosing experiences of depression may result in individuals experiencing similar social interactions to the Christian disabled community, highlighting familiar realities for individuals with disabilities and mental health issues (Swinton, 2020).

Participant stories illustrated how the problematisation of depression is deeply connected to perceptions of mental health treatment and cures in the evangelical church (Lloyd and Waller, 2020; Lloyd, 2021b). As such, the discourses surrounding depression, help-seeking and religious treatment can become pathogenic for individuals seeking support in evangelical communities. Turning to a theology of healing, this manuscript revealed the unintended consequences of a total reliance on healing for mental ill-health. Researchers have noted that healing for mental ill-health may occur when individuals develop a closer relationship with God and repent from sin and demonic influences (Dein, 2020), but this manuscript revealed the role of social relationships in all aspects of healing. Increased sociality with congregants was not only perceived to be a cure of depression, but it was also a precursor to heightened spirituality. Still, spiritualisation and healing were revealed to be a normative ideology for illness, thus shaping socio-cultural discourses on mental health in evangelical spaces. This ideology can be oppressive for many individuals and prompt them to internalise blame and ignore their potential to live well with their illness (Eiesland, 1994; Clifton, 2014). As a hallmark of evangelicalism, miraculous healing promises that individuals can be cured of physical and mental ailments and perceived as superior to biomedical treatments and alternative religious healing practices and beliefs (Gunther Brown, 2011). Gunther Brown (2011) describes that the characterisation of the evangelical God as a supernatural and ultimate healer continues to amass global followers where the recipients of divine healing believe their experiences to be a manifestation of God’s love. Illness, on the other hand, is considered sinful, demonic, and antithetical to God’s will of health and wholeness, a perception that many scholars have also documented.

Whilst this manuscript presented original insights into the construction of depression in the evangelical church using the story completion task but the nature of this method did not explicitly capture individual experiences of living with depression in an evangelical community. Future research should ethnographically capture the lived experiences of individuals with mental ill-health in United Kingdom Christian communities. The global evangelical church remains a heterogenous field for exploration, as the conceptualisation of depression and manifestation of illness and healing may vary across diverse communities. The demographic data collected in this research revealed that individuals with experiences of mental ill-health, either personal or through a friend or family member, were overwhelmingly represented in this sample. This strengthens our argument in the manuscript, as numerous stories were constructed from social interactions between individuals with mental ill-health and congregants. Though demographic variables such as gender identity and age were collected, future studies would benefit from a wider range of intersectional characteristics of research participants. Characteristics such as participants’ sexual identity, disability status, socioeconomic status, and racial and ethnic identity could reveal how individuals from diverse backgrounds experience mental health in addition to biases related to their intersectional positioning within and outside of the evangelical church.

That being said, the limitations of this research do not overshadow the novel insights gained from using story completion to understand representations of depression and help-seeking amongst evangelical communities in the United Kingdom. As Christians seek to find solace to withstand everyday distress, especially amidst the COVID-19 pandemic, the role of Christian communities becomes increasingly important in providing psychosocial support. The challenges that remain in fundamentalist communities require further critical engagement with the biopsychosocial model of depression along with trained practitioners to ensure that mental-ill health does not become exacerbated because of reductive theologies. It is then that coordinated psychosocial support of evangelical Christians can produce positive narratives of mental ill-health, whether it be learning to live well with depression or journeys toward recovery.

## Data Availability Statement

The raw data supporting the conclusions of this article will be made available by the authors, without undue reservation.

## Ethics Statement

The studies involving human participants were reviewed and approved by University of Derby. The patients/participants provided their written informed consent to participate in this study.

## Author Contributions

This project formed one component of CL’s earlier research projects in this area. CL conceptualised and designed the study, applied for ethical approval, carried out recruitment and data acquisition, provided supervision of the research project, from literature review through to study write up, wrote the methods section of the manuscript, contributed to the introduction, and edited the entire manuscript prior to submission. BM conducted qualitative analyses and the initial draft of the analysis, and introduction and discussion under supervision. GR assisted with the literature reviews and manuscript revisions for publication. All authors contributed to the manuscript revision and approved the current form of the submitted version.

## Conflict of Interest

The authors declare that the research was conducted in the absence of any commercial or financial relationships that could be construed as a potential conflict of interest.

## Publisher’s Note

All claims expressed in this article are solely those of the authors and do not necessarily represent those of their affiliated organizations, or those of the publisher, the editors and the reviewers. Any product that may be evaluated in this article, or claim that may be made by its manufacturer, is not guaranteed or endorsed by the publisher.

## Footnotes

1 It is worth noting the existence of psychological therapies which are rooted in faith, e.g., Religiously-Integrated Cognitive Behavioural Therapy (Pearce et al., 2015). Such treatments are significant as they may offer an important bridge between the often bifurcated evangelical and secular clinical settings, thus avoiding the potential pitfalls of discarding either the beneficial role of faith for wellbeing, or, psychotherapeutic intervention.
